# Prognostic Impact of High Baseline Stromal Tumor-Infiltrating Lymphocytes in the Absence of Pathologic Complete Response in Early-Stage Triple-Negative Breast Cancer

**DOI:** 10.3390/cancers14051323

**Published:** 2022-03-04

**Authors:** Nour Abuhadra, Ryan Sun, Jennifer K. Litton, Gaiane M. Rauch, Clinton Yam, Jeffrey T. Chang, Sahil Seth, Roland Bassett, Bora Lim, Alastair M. Thompson, Elizabeth Mittendorf, Beatriz E. Adrada, Senthil Damodaran, Jason White, Elizabeth Ravenberg, Rosalind Candelaria, Banu Arun, Naoto T. Ueno, Lumarie Santiago, Sadia Saleem, Sausan Abouharb, Rashmi K. Murthy, Nuhad Ibrahim, Aysegul A. Sahin, Vicente Valero, William Fraser Symmans, Debu Tripathy, Stacy Moulder, Lei Huo

**Affiliations:** 1 Department of Breast Medical Oncology, The University of Texas MD Anderson Cancer Center, Houston, TX 77030, USA; abuhadn@mskcc.org (N.A.); jlitton@mdanderson.org (J.K.L.); cyam@mdanderson.org (C.Y.); sdamodaran@mdanderson.org (S.D.); jbwhite@mdanderson.org (J.W.); eevans1@mdanderson.org (E.R.); barun@mdanderson.org (B.A.); nueno@mdanderson.org (N.T.U.); ssaleem@mdanderson.org (S.S.); sabouharb@mdanderson.org (S.A.); rmurthy1@mdanderson.org (R.K.M.); nibrahim@mdanderson.org (N.I.); vvalero@mdanderson.org (V.V.); dtripathy@mdanderson.org (D.T.); moulder_stacy@lilly.com (S.M.); 2 Department of Biostatistics, The University of Texas MD Anderson Cancer Center, Houston, TX 77030, USA; rsun3@mdanderson.org (R.S.); rlbasset@mdanderson.org (R.B.J.); 3 Department of Diagnostic Radiology, The University of Texas MD Anderson Cancer Center, Houston, TX 77030, USA; gmrauch@mdanderson.org (G.M.R.); beatriz.adrada@mdanderson.org (B.E.A.); rcandelaria@mdanderson.org (R.C.); lumarie.santiago@mdanderson.org (L.S.); 4 Department of Integrative Biology and Pharmacology, The University of Texas Health Science Center at Houston, Houston, TX 77030, USA; jeffrey.t.chang@uth.tmc.edu; 5 Department of Genomic Medicine, The University of Texas MD Anderson Cancer Center, Houston, TX 77030, USA; sseth@mdanderson.org; 6 Department of Oncology, Baylor College of Medicine, Houston, TX 77030, USA; bora.lim@bcm.edu; 7 Division of Surgical Oncology, Section of Breast Surgery, Baylor College of Medicine, Houston, TX 77030, USA; alastair.thompson@bcm.edu; 8 Division of Breast Surgery, Department of Surgery, Dana Farber/Brigham and Women’s Hospital, Boston, MA 02215, USA; emittendorf@bwh.harvard.edu; 9 Department of Pathology, The University of Texas MD Anderson Cancer Center, Houston, TX 77030, USA; asahin@mdanderson.org (A.A.S.); fsymmans@mdanderson.org (W.F.S.)

**Keywords:** triple-negative breast cancer, tumor-infiltrating lymphocytes, CD8, prognosis, neoadjuvant chemotherapy, pathologic complete response

## Abstract

**Simple Summary:**

High stromal tumor-infiltrating lymphocytes (sTILs) are associated with an improved pathologic complete response (pCR) and survival in triple-negative breast cancer (TNBC). We hypothesized that high baseline sTILs would have a favorable prognostic impact in TNBC patients without a pCR. In this study of 318 early-stage TNBC patients in a prospective clinical trial, event-free survival (EFS) in patients without a pCR was not significantly different between those with high sTILs and those with low sTILs (*p* = 0.7). Therefore, high baseline sTILs do not confer a benefit in EFS in the absence of a pCR. RNA-seq analysis predicted more CD8+ T cells in the high-sTIL group with favorable EFS compared with the high-sTIL group with unfavorable EFS, suggesting the type of lymphocytes within the TIL fraction may be an important parameter to consider for de-escalation strategies. The implications of our findings in the setting of immune checkpoint inhibitor therapy remain to be investigated.

**Abstract:**

High stromal tumor-infiltrating lymphocytes (sTILs) are associated with an improved pathologic complete response (pCR) and survival in triple-negative breast cancer (TNBC). We hypothesized that high baseline sTILs would have a favorable prognostic impact in TNBC patients without a pCR after neoadjuvant chemotherapy (NACT). In this prospective NACT study, pretreatment biopsies from 318 patients with early-stage TNBC were evaluated for sTILs. Recursive partitioning analysis (RPA) was applied to search for the sTIL cutoff best associated with a pCR. With ≥20% sTILs identified as the optimal cutoff, 33% patients had high sTILs (pCR rate 64%) and 67% had low sTILs (pCR rate 29%). Patients were stratified according to the sTIL cutoff (low vs. high) and response to NACT (pCR vs. residual disease (RD)). The primary endpoint was event-free survival (EFS), with hazard ratios calculated using the Cox proportional hazards regression model and the 3-year restricted mean survival time (RMST) as primary measures. Within the high-sTIL group, EFS was better in patients with a pCR compared with those with RD (HR 0.05; 95% CI 0.01–0.39; *p* = 0.004). The difference in the 3-year RMST for EFS between the two groups was 5.6 months (95% CI 2.3–8.8; *p* = 0.001). However, among patients with RD, EFS was not significantly different between those with high sTILs and those with low sTILs (*p* = 0.7). RNA-seq analysis predicted more CD8+ T cells in the high-sTIL group with favorable EFS compared with the high-sTIL group with unfavorable EFS. This study did not demonstrate that high baseline sTILs confer a benefit in EFS in the absence of a pCR.

## 1. Introduction 

The tumor immune microenvironment in triple-negative breast cancer (TNBC) plays a fundamental role in the response to neoadjuvant chemotherapy (NACT). Tumor-infiltrating lymphocytes (TILs) have emerged as an important immune biomarker, with multiple studies confirming their predictive and prognostic value in TNBC [1,2,3,4,5]. In the neoadjuvant setting, a pooled analysis of six neoadjuvant trials (including 906 TNBC patients) demonstrated a pathologic complete response (pCR) rate of 50% among high-stromal TIL (sTIL) (defined as ≥60%) TNBC [3]. Another pooled analysis of GeparDuo and GeparTrio trials confirmed that higher baseline TILs in pretreatment tumors are associated with higher rates of pCR, a well-established surrogate endpoint in neoadjuvant breast cancer trials [6,7,8]. A pooled analysis of nine adjuvant clinical trials (including 2148 TNBC patients) also confirmed the strong prognostic role of sTILs in early-stage TNBC patients, where patients with node-negative TNBC and ≥30% sTILs had a 3-year distant disease-free survival (dDFS) of 97% and overall survival (OS) of 99% [4]. 

The majority of studies that have validated the prognostic value of high sTILs were in the context of systemic therapy. However, recent retrospective studies have shown good outcomes in early-stage TNBC patients with high sTILs in the absence of chemotherapy [9,10]. A recent small retrospective analysis of systemically untreated stage I TNBC patients (treated at four centers; *n* = 72) showed 98% OS at 5 years in patients with >30% TILs [11]. Similarly, a retrospective analysis from Mayo Clinic of untreated TNBC patients (most with node-negative disease) demonstrated a better prognosis in lymphocyte-predominant (LP) TNBC (TILs > 50%) compared to those with lower levels of TILs [9]. In their exploratory analysis, a better iDFS was still noted when using a ≥20% cutoff for high sTILs in systemically untreated patients [9]. These findings have raised the possibility that a subset of early-stage TNBC patients with immune infiltrate-enriched tumors may have excellent outcomes without systemic therapy. There is a growing interest in the use of high sTILs to select appropriate TNBC patients for de-escalation of neoadjuvant therapy [12,13]. Better understanding of the high-sTIL TNBC population to identify those with an unfavorable outcome is equally important for optimizing clinical management. 

In this study, we aimed to evaluate the prognostic impact of high baseline sTILs in patients with early-stage TNBC receiving NACT. Our hypothesis was that high sTILs would have a favorable prognostic impact in patients who do not exhibit a pCR after NACT. To test this hypothesis, we statistically identified an optimal cutoff for high sTILs to best predict the likelihood of a pCR in our cohort and examined the impact of high sTILs on event-free survival (EFS) as the primary outcome. 

## 2. Materials and Methods

### 2.1. Patient Population 

**Figure 1:**
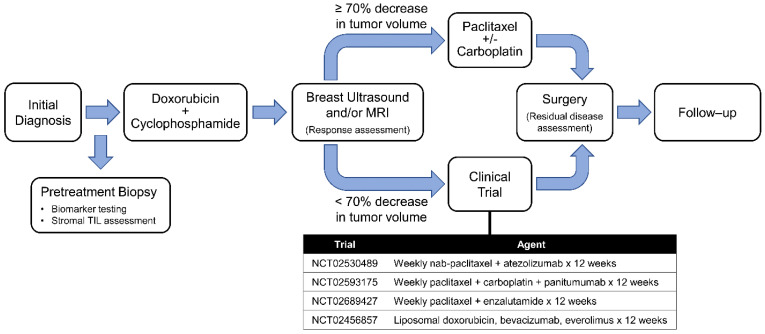
ARTEMIS trial schema. Treatment-naïve patients with localized TNBC (stages I–III) undergo a pretreatment biopsy and pretreatment ultrasound and then start 4 cycles of AC chemotherapy. Patients have their tumors imaged to assess the treatment response after AC. Patients deemed to have a chemo-sensitive disease after 4 cycles of AC are recommended to undergo standard taxane-based chemotherapy. Patients predicted to be chemo-insensitive are offered therapy on clinical trials using targeted therapy in combination with chemotherapy based on the biomarker characteristics of their tumor, a ‘second hit’ strategy in the middle of NACT to overcome chemotherapy resistance. AC, doxorubicin and cyclophosphamide; ARTEMIS, a robust TNBC evaluation framework to improve survival; NACT, neoadjuvant chemotherapy; TNBC: triple-negative breast cancer.

The ARTEMIS (**A R**obust **T**NBC **E**valuation fra**M**ework to **I**mprove **S**urvival) trial (NCT02276443) is an ongoing prospective trial and is approved by the institutional review board of MDACC. It profiles early-stage TNBC patients prior to receiving NACT and uses a unique algorithm of diagnostic imaging and biomarkers to select optimized targeted therapy trial for patients with chemotherapy-insensitive disease. Written informed consents were obtained from enrolled patients. Pretreatment (baseline) core biopsies from 318 patients with early-stage TNBC enrolled in the ARTEMIS trial, diagnosed between October 2015 and November 2019, were assessed using recursive partitioning analysis to identify the cutoff for high sTILs to best differentiate the cohort based on the association with a pCR and were included in the outcome analysis. This sTIL cutoff was applied to another 78 ARTEMIS trial patients not included in the first group for confirmation. All patients received neoadjuvant dose-dense doxorubicin (Adriamycin)/cyclophosphamide (AC). As per protocol, patients with chemotherapy-sensitive disease (≥70% tumor volumetric reduction by ultrasound after 4 cycles of AC) went on to receive taxane-based NACT. Patients with chemotherapy-insensitive disease (<70% tumor volumetric reduction by ultrasound after 4 cycles of AC) were offered enrollment in a phase II clinical trial with a standard chemotherapy backbone and targeted agent based on the patients’ biomarker profiling (Figure 1). Because this study aimed to focus on the impact of high sTILs on the response to standard NACT, patients with a pCR in phase II clinical trials (*n* = 14, including 6 with high sTILs and 8 with low sTILs) during the same enrollment period for this study were excluded from both the recursive partitioning analysis and the outcomes analysis. This step was taken because it was impossible to distinguish whether the pCR was due to the effect of the targeted agents or due to the chemotherapy backbone. 

### 2.2. Pathological Evaluation 

TNBC was defined as a tumor that was estrogen receptor (ER) negative, progesterone receptor (PR) negative, and human epidermal growth factor receptor 2 (HER2) negative. In the ARTEMIS trial, ER and PR negativity was defined as <10% staining of invasive carcinoma cells of any intensity by immunohistochemistry. HER2 negativity was defined according to the American Society of Clinical Oncology/College of American Pathologists HER2 testing guidelines [14]. Stromal TILs were assessed on hematoxylin and eosin (H&E)-stained baseline core biopsy slides based on the International TIL Working Group guidelines, whereby the percentage of stromal TILs is calculated as the area of the tumor stroma occupied by mononuclear inflammatory cells divided by the total tumor stromal area [15]. Stromal TILs were evaluated by a central pathology review and recorded in increments of 10%, with 5% and above rounded up to the next higher increment (Supplementary Figure S1). A pCR was defined as the absence of invasive cancer within the breast and regional lymph nodes (ypT0/ypN0) and residual disease (RD) as the lack of a pCR. Residual cancer burden (RCB) indices and class were calculated according to previously published methods from the histopathological features of residual disease specimens after NACT [16,17]. 

### 2.3. Recursive Partitioning Analysis 

We applied recursive partitioning analysis to search for the sTIL cutoff that was best associated with a pCR in our study. Briefly, recursive partitioning analysis is a tree-based method that splits the population according to one or more explanatory factors so that each subgroup demonstrates similar outcomes. In this setting, the only explanatory factor was sTILs assessed as a continuous variable and the outcome was a pCR. So the recursive partitioning analysis selected the level of sTILs that would best predict subjects who would achieve a pCR on following the ARTEMIS treatment protocol. After the sTIL threshold was set, with subjects meeting the threshold categorized into the high-sTIL group, we correlated the sTIL groups with outcomes.

### 2.4. Outcomes Analysis 

The primary endpoint for the study was EFS, defined as the time from the date of diagnosis to the date of the first relapse (locoregional or distant metastatic disease) or death resulting from any cause, whichever occurred first. The secondary endpoint was OS, defined as the time from the date of diagnosis to the date of death from any cause. Patients who were alive (for OS) and event free (for EFS) were censored at the date of last follow-up. We calculated the 3-year restricted mean survival time (RMST) and fitted Cox proportional hazards regression models for each of the outcomes. The latter was not performed if any of the arms did not have an event. Patients were stratified according to sTILs (cutoff identified by recursive partitioning analysis) and pCR versus RD. 

### 2.5. Additional Statistical Analysis

Wilcoxon rank-sum tests were used to compare the distribution of stromal TILs between prognostic groups. Fisher’s exact tests were used to compare the distribution of categorical parameters between groups. All statistical tests used a significance level of 5%. No adjustments for multiple testing were made. All statistical analyses were performed using R version 4.1.1.

### 2.6. Molecular/Vanderbilt Subtype Analysis 

Gene expression profiles of the tumors were obtained using Affymetrix U133 microarrays and preprocessed with robust multiarray analysis (RMA) [18]. The data were quantile normalized and uploaded to the TNBCType website for subtype estimation [19].

### 2.7. Whole Transcriptomic Sequencing (RNA-Seq)

Total RNA was isolated, quality-checked using a 2200 Tapestation (Agilent Technologies), and converted to cDNA using the Ovation RNA-Seq System V2 (NuGEN). A library was prepared using the KAPA library prep kit. Exomes were captured using the Roche Nimblegen (Exome V3) probes and then sequenced on a NovaSeq 6000 (Illunima). From the sequence data, we estimated the transcripts per million using RSEM [20], with a STAR aligner [21]. We predicted the immune cell population using CIBERSORT [22] in absolute mode so that the scores can be compared across cell types.

## 3. Results

### 3.1. Identifying an Optimal Cutoff for High sTILs 

**Figure 2:**
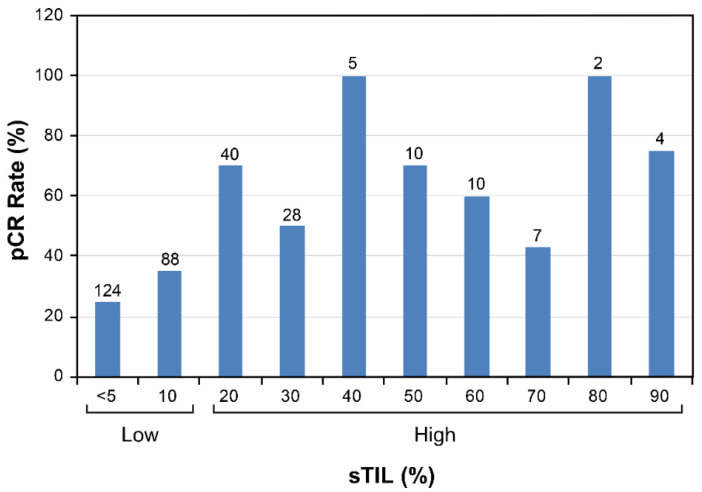
The pCR rates in sTIL groups in 10% increments in 318 patients. Recursive partitioning analysis identified ≥20% for defining high sTILs in association with a pCR. The total number of patients in each sTIL group is shown on top of the corresponding column. pCR, pathologic complete response; sTIL, stromal tumor-infiltrating lymphocyte.

Among the 318 patients included in this study, 130 (41%) exhibited a pCR. Using recursive partitioning analysis, we identified 20% (i.e., low < 20% and high ≥ 20%) as the optimal cutoff for defining high baseline sTILs in association with a pCR (Figure 2). With this cutoff applied, the pCR rate was 64% (68/106) in the high-sTIL group and 29% (62/212) in the low-sTIL group. We further tested the cutoff in 78 additional patients enrolled in the ARTEMIS trial whose data subsequently became available. In this group, the pCR rate was 60% (*n* = 17/28) in the high-sTIL subset and 30% (16/50) in the low-sTIL subset, similar to those observed with patients included in the recursive partitioning analysis. 

### 3.2. Patient Baseline Characteristics 

Clinicopathological characteristics of the 318 patients grouped with the 20% sTIL cutoff are summarized in Table 1. Compared with the low-sTIL group, the high-sTIL group showed a significantly higher pCR rate, a lower frequency of enrollment in phase II neoadjuvant clinic trials and adjuvant systemic therapy, a higher rate of grade 3 disease, and a different distribution in the tumor stage and the nodal stage. Among patients with RD, 47% (89/188) received adjuvant systemic therapy, including 70% (62/89) patients who received capecitabine.

### 3.3. Prognostic Significance of High sTILs 

**Figure 3:**
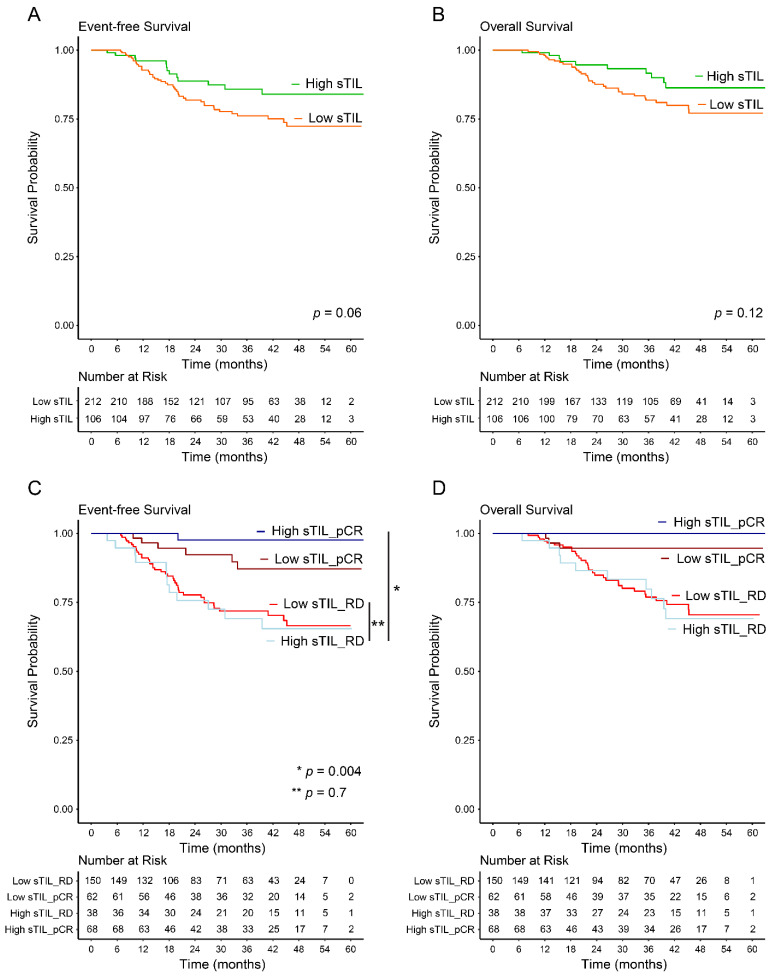
Event-free survival (EFS) and overall survival (OS) in high-sTIL and low-sTIL patients. (**A**) Kaplan–Meier plots of EFS between the overall-high-sTIL and overall-low-sTIL groups. (**B**) Kaplan–Meier plots of OS between the overall-high-sTIL and overall-low-sTIL groups. (**C**) Kaplan–Meier plots of EFS between the high-sTIL and low-sTIL groups, with a pCR and with RD. (**D**) Kaplan–Meier plots of OS between the high-sTIL and low-sTIL groups, with a pCR and with RD. pCR, pathologic complete response; RD, residual disease; sTIL, stromal tumor-infiltrating lymphocyte.

The median follow-up period was 36.5 months (range 4.2–62.9 months). Nineteen (6%) patients experienced a locoregional recurrence, 49 (15%) patients had a distant recurrence, and 44 (14%) patients died. We evaluated EFS and OS in the overall high-sTIL versus low-sTIL groups regardless of the pCR status. There was a trend of EFS in the overall-high-sTIL group when compared with the overall-low-sTIL group (HR 0.57; 95% CI 0.30–1.03; *p* = 0.06) (Figure 3A). OS did not reach statistical significance (*p* = 0.12; Figure 3B). The 3-year EFS was 86% for the high-TIL group and 76% for the low-TIL group, and the 3-year OS was 92% for the high-TIL group and 82% for the low-TIL group. We also evaluated EFS and OS in the sTIL groups that exhibited a pCR and those with RD. Among patients with high baseline sTILs, there was a significant difference in EFS between those who achieved a pCR compared with those with RD (HR 0.05; 95% CI 0.01–0.39; *p* = 0.004) (Figure 3C). The difference in the 3-year restricted mean EFS time in these two groups was 5.6 months (95% CI 2.3–8.8; *p* = 0.001); i.e., over 3 years of follow-up, high-sTIL patients who achieved a pCR would be expected to survive event free for an average of 5.6 months longer than high-sTIL patients with RD. Although a Cox proportional hazards regression model was not fitted for OS because one arm did not have any events (Figure 3D), a difference in the 3-year restricted mean OS time in these two groups was observed, which showed that over 3 years of follow-up, high-sTIL patients who achieved a pCR would be expected to have a longer OS by 3.3 months than high-sTIL patients with RD (95% CI 0.8–5.8; *p* = 0.01). In contrast, among patients who had RD, there was not a significant difference in EFS or OS between those with high baseline sTILs and those with low baseline sTILs (*p* = 0.7 each; Figure 3C,D). RMST analysis also showed a similar 3-year restricted mean EFS time and a similar 3-year restricted mean OS time between these two groups (*p* = 0.7 and *p* = 0.9, respectively). 

### 3.4. Prognostic Indicators in the High-sTIL Patients

With the findings of the prognostic difference within the high-sTIL patients, we evaluated the association between a response to a neoadjuvant treatment and EFS by RCB categories. In the high-sTIL patients (*n* = 106), those with RCB I (*n* = 11) had favorable EFS similar to those with RCB 0, while those with RCB II and RCB III had worse EFS compared with RCB 0 and RCB I patients (*p* < 0.001; Supplementary Figure S2). Subsequent analysis of the high-sTIL patients was performed to compare between those with RCB 0 and I, which were considered as the group with favorable EFS, and those with RCB II and III, considered as the group with unfavorable EFS.

Molecular subtyping (Vanderbilt subtypes) was performed on 67 of the 106 patients, where we were able to obtain high-quality gene expression profiles [19,23]. The immunomodulatory (IM) subtype was more frequent in the RCB 0/I group (21/46; 46%) compared with the RCB II/III group (7/21; 33%), consistent with a model that links high immune activation with good outcomes. In contrast, the basal-like 2 (BL2) subtype and the luminal androgen receptor (LAR) subtype were more frequent in the RCB II/III group (14% versus 4% for BL2 and 19% versus 9% for LAR). However, the difference in the proportions of the subtypes between the two prognostic groups did not reach statistical significance (Table 2).

**Figure 4:**
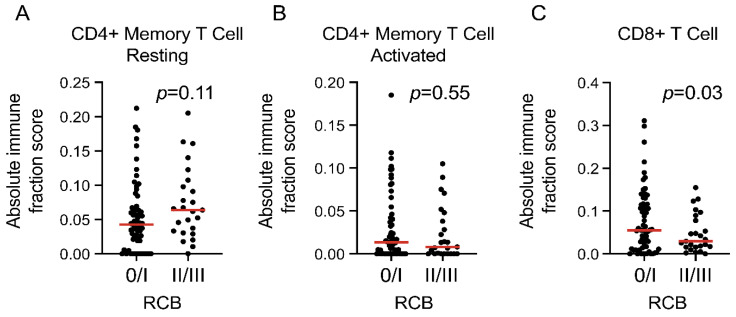
Comparison of T cells by whole transcriptomic sequencing (RNA-seq) between RCB 0/I (*n* = 64) and RCB II/III (*n* = 25) groups in high-sTIL patients. (**A**) resting CD4+ memory T cells; (**B**) activated CD4+ memory T cells; (**C**) CD8+ T cells. The horizontal bar in each plot represents the median of the scores. RCB, residual cancer burden; sTIL, stromal tumor-infiltrating lymphocytes.

The amount and composition of the lymphocytes associated with tumor in the high-sTIL patients were examined. First, the amount of sTILs (ranging from 20% to 90% in 10% increments) was compared between the RCB 0/I and the RCB II/III groups. There was no statistically significant difference in the sTILs as a continuous variable between the two groups (*p* = 0.78). When a cutoff of 30% was used to dichotomize the cohort to low (20% and 30% sTILs) and high (>30%), there was no significant difference between the two groups (*p* = 0.49; Table 2). A cutoff of 20% was applied with similar results (*p* = 1.00). Thus, in the high-sTIL patients, the relative abundance of sTILs was not associated with EFS based on the RCB groups described here. Next, to determine whether specific types of lymphocytes may be associated with EFS, CIBERSORT (absolute mode [24]) was applied to whole transcriptomic sequencing (RNA-seq) data (*n* = 89) to predict the abundance of CD4+ and CD8+ T cells as a fraction of the total cell population. The results revealed no significant difference in resting CD4+ memory T cells or activated CD4+ memory T cells between the RCB 0/I (*n* = 64) and RCB II/III (*n* = 25) groups (*p* = 0.11 and 0.55, respectively) but predicted more CD8+ T cells in the RCB 0/I group compared with the RCB II/III group (*p* = 0.03; Figure 4). No naïve CD4+ T cells were detected, possibly due to a lack of sensitivity in the computational algorithm.

## 4. Discussion

In this study, we assessed the prognostic significance of high baseline sTILs in patients with early-stage TNBC treated with NACT in a prospective trial. Our results show that in this setting, high sTILs do not confer a benefit in EFS in the absence of a pCR, thus providing a counterargument to our original hypothesis. Many studies have evaluated the prognostic value of TILs in early-stage TNBC; however, many of the data available were retrospective extrapolations from multiple randomized trials and meta-analyses. The seminal work by Dieci et al. was one of the earliest to suggest adopting TILs as a stratification parameter in clinical trials for TNBC and suggested an interesting possibility of withholding adjuvant chemotherapy for those with early-stage TNBC and high TILs [1]. Leon-Ferre et al. evaluated the prognostic impact of TILs in early TNBC and included patients who had never received adjuvant chemotherapy, demonstrating that TILs were independently associated with iDFS and OS and that they remained prognostic in systemically untreated patients [9]. These findings suggest an intrinsically favorable role of TILs in TNBC whether chemotherapy is administered or not. A similar retrospective study evaluating prognosis in untreated patients identified a subset of stage I TNBC patients with high sTILs that had an excellent prognosis without adjuvant chemotherapy [11]. However, the authors acknowledged the limitations of that work given the retrospective nature and inevitable selection bias (e.g., cohort enriched for patients who were not good candidates for chemotherapy or patients who were not offered chemotherapy based on favorable pathologic features). In our cohort, of all patients treated with NACT, only 12% had stage I TNBC. Therefore, the question of omitting neoadjuvant or adjuvant chemotherapy in stage I TNBC with high sTILs is yet to be answered with prospective analysis of a larger sample size. In our study, we have shown a significant difference in EFS between high-sTIL patients showing a pCR and high-sTIL patients with RD. When we evaluated the EFS in high- and low-sTIL patients irrespective of the pCR status, there was a trend for favorable EFS in the high-sTIL group compared with the low-sTIL group (*p* = 0.06). These findings suggest that the prognostic impact of high sTILs that has been noted in prior retrospective studies may be a function of the improved pCR rates associated with this subset of patients.

We examined additional phenotypic characteristics that could account for the significant difference in EFS among the high-sTIL patients, including Vanderbilt subtypes and immune cell composition. Interestingly, analysis of the RNA-seq data predicted significantly more CD8+ T cells in the RCB 0/I group compared with the RCB II/III group, suggesting that the role of high sTILs as a prognostic marker may be refined by more precise probing of the lymphocyte population. Thus, when selecting early-stage TNBC patients for de-escalation strategies, the type of lymphocytes within the TIL fraction may be an important parameter to consider in addition to the amount of sTILs. While this finding is consistent with prior literature, showing that increased CD8+ T cell infiltration is associated with better survival in TNBC [25,26,27,28], to the best of our knowledge, ours is the first prospective study to provide the evidence in the high-sTIL patient group. Additional ongoing work on the ARTEMIS cohort to elucidate the phenotypic characteristics of the tumor immune cells may shed light upon the mechanisms of chemoresistance and disease recurrence within the high-sTIL group, providing opportunities to further refine individual treatment strategies.

Seminal studies on the predictive and prognostic role of TILs have evaluated them as a continuous parameter (in increments of 10) as well as using different assigned cutoffs to define lymphocyte-predominant breast cancer (LPBC), including ≥30, ≥50, and ≥60% [1,3,29]. The variation in the cutoff for high TILs applied in the literature, despite recommendations published by the International TIL Working Group focused on standardizing TIL evaluation [15,30], may be in part due to how TILs have been assessed in various studies. As an example, in the analysis of the prognostic and predictive value of TILs using patients from the BIG 02-98 trial, LPBC was defined as >50% infiltration of either stromal or intratumoral lymphocytes [29], whereas others only included sTILs [2,3]. When sTILs and intratumoral TILs (itTILs) have been evaluated, several reports showed a significant correlation between the two measures, as well as improved outcomes in TNBC with both sTILs and itTILs [1,8,29,31]. Because sTILs appear to be more reproducible, they have emerged as a preferred marker on H&E-stained slides and selected as the primary measure in this study [15,32]. Another factor that may contribute to the inconsistency in the reported TIL cutoff is the type of pathology material used to assess TILs (large surgical sections versus core biopsies versus tissue microarrays), because scores may be affected by sample size. In addition, particular morphologic features of the tumor area, such as heterogeneous distribution of lymphocytes in the tumor stroma, may introduce interobserver variation during TIL assessment. In our study, we used recursive partitioning statistical analysis to find a cutoff ≥20%, which defined 33% (106/318) of the cohort as high sTIL based on the best association with pCR prediction. Interestingly, other studies that have reported high sTILs as a favorable predictive/prognostic factor in TNBC grouped 30–35% of their cohorts as high sTIL, albeit with different cutoff numbers [2,3,4]. These findings suggest that even though finding a uniform cutoff between different cohorts is challenging due to aforementioned reasons, about one-third of TNBC patients have high sTILs in association with treatment response or outcome, which could be used as a rule of thumb for TIL evaluation in predictive and prognostic studies. The cutoff identified by recursive partitioning analysis in our study allowed us to dichotomize the cohort to conduct the outcome analysis and could be applied to future work involving sTIL assessment with the ARTEMIS cohort.

Although this study included a relatively large cohort of TNBC patients, some subgroups may have a small number of subjects, which is a limitation of the study. For example, the number of patients with high sTILs and without a pCR was relatively small (*n* = 38). With the small sample size and limited number of events, the analysis may be underpowered. Repeating the analysis with more patients is needed to confirm our findings. Nonetheless, our data reflect the well-established body of literature demonstrating higher pCR rates associated with high sTILs in TNBC patients. Another limitation is the relatively short follow-up time; however, the results remain clinically meaningful given that the highest risk of recurrence in TNBC is within the first 3 years [33]. Longer follow-up is required to determine how later events affect differences in EFS and OS based on sTILs as these data mature.

In this single-institution study, we evaluated the prognostic significance of high baseline sTILs in patients with early-stage TNBC treated with NACT. In addition to the prospective nature of this study, all data were collected in the context of an ongoing clinical trial. In summary, we statistically identified a cutoff for high sTILs for selecting patients with an increased likelihood of a pCR after anthracycline-based NACT. We demonstrated a significant difference in EFS between high sTILs/pCR and high sTILs/RD. These findings highlight that while high sTILs are an important immunologic biomarker, they may not be sufficient as a stand-alone biomarker when considering de-escalation strategies. Our results suggest that high baseline sTILs do not confer an EFS benefit in patients in the absence of a pCR after NACT; therefore, high-sTIL patients with RD should be treated as per standard care. If we are able to further stratify high-sTIL patients with RD into favorable and unfavorable prognostic subgroups based on other factors, these patients could be treated more selectively with adjuvant therapy. Additionally, with the recent approval of neoadjuvant pembrolizumab for TNBC [34], the implications of our findings in the setting of immune checkpoint inhibitor therapy remain to be investigated. More work is needed to identify additional clinical and pathological parameters to enhance the prognostic role of high sTILs in early-stage TNBC.

## Figures and Tables

**Table 1:** Patient clinicopathologic characteristics.

	Overall	Low TILs (<20%)	High TILs (≥20%)	*p*-Value
Total Patients (%)	318 (100)	212	106	
Median Age at Diagnosis (year range)	52.5 (24–77)	54 (24–77)	49 (27–77)	
**Race** [*n* (%)]				0.087
White	240 (75)	159 (75)	81 (76)	
Black	51 (16)	39 (18)	12 (11)	
Asian	25 (8)	13 (6)	12 (11)	
Native Hawaiian or Other Pacific Islander	1 (0.5)	1 (1)	0	
Other	1 (0.5)	-	1 (1)	
**Tumor Stage** [*n* (%)]				**0.002**
T1	60 (19)	34 (16)	26 (24)	
T2	208 (65)	134 (63)	74 (70)	
T3	37 (12)	33 (15)	4 (4)	
T4	13 (4)	11(5)	2 (2)	
**Nodal Stage** [*n* (%)]				**0.009**
N0	190 (60)	132 (62)	58 (55)	
N1	79 (25)	46 (22)	33 (31)	
N2	8 (3)	2 (1)	6 (6)	
N3	41 (12)	32 (15)	9 (8)	
**Clinical TNM Stage** [*n* (%)]				0.315
I	38 (12)	25 (12)	13 (12)	
II	210 (66)	135 (64)	75 (71)	
III	70 (22)	52 (24)	18 (17)	
**Histologic Grade** [*n* (%)]				**0.019**
1	2 (0.5)	2 (1)	0	
2	38 (12)	32 (15)	6 (6)	
3	278 (87.5)	178 (84)	100 (94)	
**Histologic Type** [*n* (%)]				0.473
Invasive ductal	265 (83)	172 (81)	93 (88)	
Invasive lobular	3 (1)	2 (1)	1 (1)	
Metaplastic	35 (11)	26 (12)	9 (8)	
Other *	15 (5)	12 (6)	3 (3)	
**Systemic Neoadjuvant Therapy** ^†^ [*n* (%)]				**0.009**
Standard chemotherapy	267 (84)	168 (79)	99 (93)	
Phase II NAT clinical trial ^††^	51 (16)	44 (21)	7 (7)	
**Surgery** [*n* (%)]				0.502
Mastectomy	126 (40)	89 (42)	37 (35)	
Breast conserving surgery	187 (59)	120 (57)	67 (63)	
No surgery (due to progression)	5 (1)	3 (2)	2 (2)	
**Pathologic Response** [*n* (%)]				**<0.001**
pCR/RCB-0	130 (41)	62 (29)	68 (64)	
RCB I	45 (14)	34 (16)	11 (10)	
RCB-II	109 (34)	92 (43)	17 (16)	
RCB-III	34 (11)	24 (11)	10 (10)	
**Adjuvant Radiation** [*n* (%)]				0.500
Yes	251 (79)	168 (79)	83 (78)	
No	64 (20)	43 (20)	21 (20)	
Unknown (lost to follow-up)	3 (1)	1 (1)	2 (2)	
**Adjuvant Systemic Therapy** ^‡^ [*n* (%)]				**<0.001**
Yes	89 (28)	74 (34)	15 (14)	
No	226 (71)	137 (65)	89 (84)	
Unknown (lost to follow-up)	3 (1)	1 (1)	2 (2)	

* Includes invasive mammary, apocrine, epithelioid, and neuroendocrine. † Patients with a pCR in experimental therapy were excluded from the analysis. Shown here are patients with residual disease after experimental therapy with a standard chemotherapy backbone. †† Phase II trial of neoadjuvant nab-paclitaxel and atezolizumab (NCT02530489); *n* = 14. Phase II trial of neoadjuvant liposomal doxorubicin, bevacizumab, and everolimus (DAT) in TNBC insensitive to standard neoadjuvant chemo (NCT02456857); *n* = 15. Phase II of panitumumab, carboplatin, and paclitaxel (PaCT) in localized TNBC insensitive to NACT (NCT02593175); *n* = 11. Phase IIB neoadjuvant enzalutamide plus paclitaxel for AR+ TNBC (NCT02689427); *n* = 11. ‡ Adjuvant systemic therapy including endocrine therapy for patients with low ER+ after pretreatment or surgical pathology; HER2-directed therapy in clinical trial for HER2 1+ IHC in baseline biopsy or HER2+ on the surgical specimen; capecitabine for patients with residual disease following CREATE-X results reported; immunotherapy (atezolizumab or pembrolizumab) in the clinical trial; adjuvant taxane.

**Table 2:** Comparison of the molecular/Vanderbilt subtype and stromal-tumor-infiltrating lymphocytes (sTILs) between residual cancer burden (RCB) 0/I and RCB II/III groups in high-sTIL patients.

Variable	Category	RCB 0/I *n* (%)	RCB II/III *n* (%)	Total *n* (%)	*p*-Value
**Vanderbilt Subtype**	BL1	12 (26)	5 (24)	17 (25)	0.62
	BL2	2 (4)	3 (14)	5 (8)	
	IM	21 (46)	7 (33)	28 (42)	
	LAR	4 (9)	4 (19)	8 (12)	
	MSL	1 (2)	0 (0)	1 (1)	
	M	1 (2)	0 (0)	1 (1)	
	UNS	5 (11)	2 (10)	7 (10)	
	Total	46	21	67	
**sTIL**	20%/30%	49 (62)	19 (70)	68 (64)	0.49
	>30%	30 (38)	8 (30)	38 (36)	
	Total	79	27	106	

BL1, basal-like 1; BL2, basal-like 2; IM, immunomodulatory; LAR, luminal androgen receptor; M, mesenchymal; MSL, mesenchymal stem-like; UNS, unstable.

## Data Availability

The data that support the findings of this study are available from the corresponding author upon reasonable request.

## Supplementary Materials

The following supporting information can be downloaded at: https://www.mdpi.com/article/10.3390/cancers14051323/s1. Figure S1: Photomicrographs of high sTIL and low sTIL tumors. Figure S2: Kaplan-Meier plots of event-free survival in high sTIL patients based on RCB groups.

## References

[1] Dieci M.V., Mathieu M.C., Guarneri V., Conte P., Delaloge S., Andre F., Goubar A. (2015). Prognostic and predictive value of tumor-infiltrating lymphocytes in two phase III randomized adjuvant breast cancer trials. Ann. Oncol..

[2] Herrero-Vicent C., Guerrero A., Gavilá J., Gozalbo F., Hernández A., Sandiego S., Algarra M.A., Calatrava A., Guillem-Porta V., Ruiz-Simón A. (2017). Predictive and prognostic impact of tumour-infiltrating lymphocytes in triple-negative breast cancer treated with neoadjuvant chemotherapy. Ecancermedicalscience.

[3] Denkert C., von Minckwitz G., Darb-Esfahani S., Lederer B., Heppner B.I., Weber K.E., Budczies J., Huober J., Klauschen F., Furlanetto J. (2018). Tumour-infiltrating lymphocytes and prognosis in different subtypes of breast cancer: A pooled analysis of 3771 patients treated with neoadjuvant therapy. Lancet Oncol..

[4] Loi S., Drubay D., Adams S., Pruneri G., Francis P.A., Lacroix-Triki M., Joensuu H., Dieci M.V., Badve S., Demaria S. (2019). Tumor-Infiltrating Lymphocytes and Prognosis: A Pooled Individual Patient Analysis of Early-Stage Triple-Negative Breast Cancers. J. Clin. Oncol..

[5] Gao G., Wang Z., Qu X., Zhang Z. (2020). Prognostic value of tumor-infiltrating lymphocytes in patients with triple-negative breast cancer: A systematic review and meta-analysis. BMC Cancer.

[6] Prowell T.M., Pazdur R. (2012). Pathological complete response and accelerated drug approval in early breast cancer. N. Engl. J. Med..

[7] Cortazar P., Zhang L., Untch M., Mehta K., Costantino J.P., Wolmark N., Bonnefoi H., Cameron D., Gianni L., Valagussa P. (2014). Pathological complete response and long-term clinical benefit in breast cancer: The CTNeoBC pooled analysis. Lancet.

[8] Denkert C., Loibl S., Noske A., Roller M., Müller B.M., Komor M., Budczies J., Darb-Esfahani S., Kronenwett R., Hanusch C. (2010). Tumor-associated lymphocytes as an independent predictor of response to neoadjuvant chemotherapy in breast cancer. J. Clin. Oncol..

[9] Leon-Ferre R.A., Polley M.-Y., Liu H., Gilbert J.A., Cafourek V., Hillman D.W., Elkhanany A., Akinhanmi M., Lilyquist J., Thomas A. (2018). Impact of histopathology, tumor-infiltrating lymphocytes, and adjuvant chemotherapy on prognosis of triple-negative breast cancer. Breast Cancer Res. Treat..

[10] Park J.H., Lee H.J., Lee S.B., Ahn J.-H., Kim J.E., Jung K.H., Gong G., Son B.-H., Ahn S.-H., Kim S.-B. (2019). Intrinsic Prognostic Impact of Tumor-infiltrating Lymphocytes in Systemically Untreated Patients with Early-stage Triple-negative Breast Cancer. Anticancer Res..

[11] Park J.H., Jonas S.F., Bataillon G., Criscitiello C., Salgado R., Loi S., Viale G., Lee H.J., Dieci M.V., Kim S.-B. (2019). Prognostic value of tumor-infiltrating lymphocytes in patients with early-stage triple-negative breast cancers (TNBC) who did not receive adjuvant chemotherapy. Ann. Oncol..

[12] Carey L.A. (2017). De-escalating and escalating systemic therapy in triple negative breast cancer. Breast.

[13] Curigliano G., Burstein H.J., Winer E.P., Gnant M., Dubsky P., Loibl S., Colleoni M., Regan M., Piccart-Gebhart M., Senn H.-J. (2019). De-escalating and escalating treatments for early-stage breast cancer: The St. Gallen International Expert Consensus Conference on the Primary Therapy of Early Breast Cancer 2017. Ann. Oncol..

[14] Wolff A.C., Hammond M.E.H., Hicks D.G., Dowsett M., McShane L.M., Allison K.H., Allred D.C., Bartlett J.M., Bilous M., Fitzgibbons P. (2014). Recommendations for human epidermal growth factor receptor 2 testing in breast cancer: American Society of Clinical Oncology/College of American Pathologists clinical practice guideline update. Arch. Pathol. Lab. Med..

[15] Salgado R., Denkert C., Demaria S., Sirtaine N., Klauschen F., Pruneri G., Wienert S., Van den Eynden G., Baehner F.L., Penault-Llorca F. (2015). The evaluation of tumor-infiltrating lymphocytes (TILs) in breast cancer: Recommendations by an International TILs Working Group 2014. Ann. Oncol..

[16] Symmans W.F., Peintinger F., Hatzis C., Rajan R., Kuerer H., Valero V., Assad L., Poniecka A., Hennessy B., Green M. (2007). Measurement of residual breast cancer burden to predict survival after neoadjuvant chemotherapy. J. Clin. Oncol..

[17] Symmans W.F., Wei C., Gould R., Yu X., Zhang Y., Liu M., Walls A., Bousamra A., Ramineni M., Sinn B. (2017). Long-Term Prognostic Risk after Neoadjuvant Chemotherapy Associated with Residual Cancer Burden and Breast Cancer Subtype. J. Clin. Oncol..

[18] Irizarry R.A., Hobbs B., Collin F., Beazer-Barclay Y.D., Antonellis K.J., Scherf U., Speed T. (2003). Exploration, normalization, and summaries of high density oligonucleotide array probe level data. Biostatistics.

[19] Chen X., Li J., Gray W.H., Lehmann B.D., Bauer J.A., Shyr Y., Pietenpol J.A. (2012). TNBCtype: A Subtyping Tool for Triple-Negative Breast Cancer. Cancer Inform..

[20] Li B., Ruotti V., Stewart R.M., Thomson J.A., Dewey C.N. (2010). RNA-Seq gene expression estimation with read mapping uncertainty. Bioinformatics.

[21] Dobin A., Davis C.A., Schlesinger F., Drenkow J., Zaleski C., Jha S., Batut P., Chaisson M., Gingeras T.R. (2013). STAR: Ultrafast universal RNA-seq aligner. Bioinformatics.

[22] Newman A.M., Liu C.L., Green M.R., Gentles A.J., Feng W., Xu Y., Hoang C.D., Diehn M., Alizadeh A.A. (2015). Robust enumeration of cell subsets from tissue expression profiles. Nat. Methods.

[23] Lehmann B.D., Bauer J.A., Chen X., Sanders M.E., Chakravarthy A.B., Shyr Y., Pietenpol J.A. (2011). Identification of human triple-negative breast cancer subtypes and preclinical models for selection of targeted therapies. J. Clin. Investig..

[24] Chen B., Khodadoust M.S., Liu C.L., Newman A.M., Alizadeh A.A. (2018). Profiling Tumor Infiltrating Immune Cells with CIBERSORT. Methods Mol. Biol..

[25] Mahmoud S.M.A., Paish E.C., Powe D.G., Macmillan R.D., Grainge M.J., Lee A.H.S., Ellis I., Green A. (2011). Tumor-infiltrating CD8+ lymphocytes predict clinical outcome in breast cancer. J. Clin. Oncol..

[26] Vihervuori H., Autere T.A., Repo H., Kurki S., Kallio L., Lintunen M.M., Talvinen K., Kronqvist P. (2019). Tumor-infiltrating lymphocytes and CD8+ T cells predict survival of triple-negative breast cancer. J. Cancer Res. Clin. Oncol..

[27] Oshi M., Asaoka M., Tokumaru Y., Yan L., Matsuyama R., Ishikawa T., Endo I., Takabe K. (2020). CD8 T cell score as a prognostic biomarker for triple negative breast cancer. Int. J. Mol. Sci..

[28] Byrne A., Savas P., Sant S., Li R., Virassamy B., Luen S.J., Beavis P., Mackay L.K., Neeson P.J., Loi S. (2020). Tissue-resident memory T cells in breast cancer control and immunotherapy responses. Nat. Rev. Clin. Oncol..

[29] Loi S., Sirtaine N., Piette F., Salgado R., Viale G., Van Eenoo F., Rouas G., Francis P., Crown J.P., Hitre E. (2013). Prognostic and predictive value of tumor-infiltrating lymphocytes in a phase III randomized adjuvant breast cancer trial in node-positive breast cancer comparing the addition of docetaxel to doxorubicin with doxorubicin-based chemotherapy: BIG 02-98. J. Clin. Oncol..

[30] Dieci M.V., Radosevic-Robin N., Fineberg S., van den Eynden G., Ternes N., Penault-Llorca F., Pruneri G., D’Alfonso T.M., Demaria S., Castaneda C. (2018). Update on tumor-infiltrating lymphocytes (TILs) in breast cancer, including recommendations to assess TILs in residual disease after neoadjuvant therapy and in carcinoma in situ: A report of the International Immuno-Oncology Biomarker Working Group on Breast Cancer. Semin. Cancer Biol..

[31] Issa-Nummer Y., Darb-Esfahani S., Loibl S., Kunz G., Nekljudova V., Schrader I., Sinn B.V., Ulmer H.-U., Kronenwett R., Just M. (2013). Prospective validation of immunological infiltrate for prediction of response to neoadjuvant chemotherapy in HER2-negative breast cancer—A substudy of the neoadjuvant GeparQuinto trial. PLoS ONE.

[32] Kos Z., Roblin E., Kim R.S., Michiels S., Gallas B.D., Chen W., Van de Vijver K., Goel S., Adams S., Demaria S. (2020). Pitfalls in assessing stromal tumor infiltrating lymphocytes (sTILs) in breast cancer. NPJ Breast Cancer.

[33] Pogoda K., Niwińska A., Murawska M., Pieńkowski T. (2013). Analysis of pattern, time and risk factors influencing recurrence in triple-negative breast cancer patients. Med. Oncol..

[34] Schmid P., Cortes J., Pusztai L., McArthur H., Kümmel S., Bergh J., Denkert C., Park Y.H., Hui R., Harbeck N. (2020). Pembrolizumab for Early Triple-Negative Breast Cancer. N. Engl. J. Med..

