# Supplementary material for: Prognostic Impact of High Baseline Stromal Tumor-Infiltrating Lymphocytes in the Absence of Pathologic Complete Response in Early-Stage Triple-Negative Breast Cancer
Source: Cancers (Basel). 2022 Mar 4;14(5):1323. doi: 10.3390/cancers14051323 (PMC8909018; doi:10.3390/cancers14051323)
Supplement: Supplementary file 1 [file cancers-14-01323-s001.zip › cancers-1596627-supplementary.pdf]

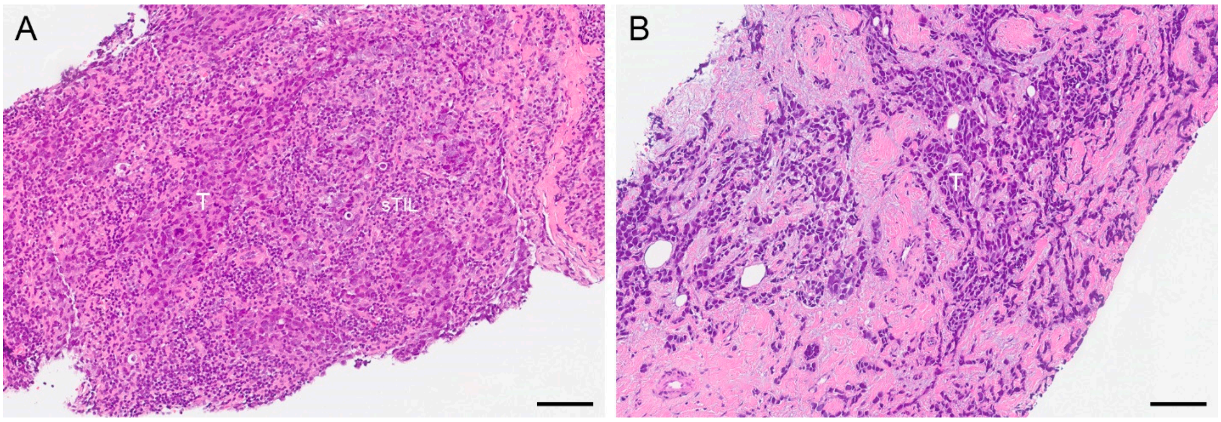

Supplementary Figure S1. Photomicrographs of high sTIL and low sTIL tumors. (A) A tumor with high sTIL. (B) A tumor with low sTIL. T, tumor. Scale bar, 100 μm.
sTIL, stromal tumor-infiltrating lymphocytes.

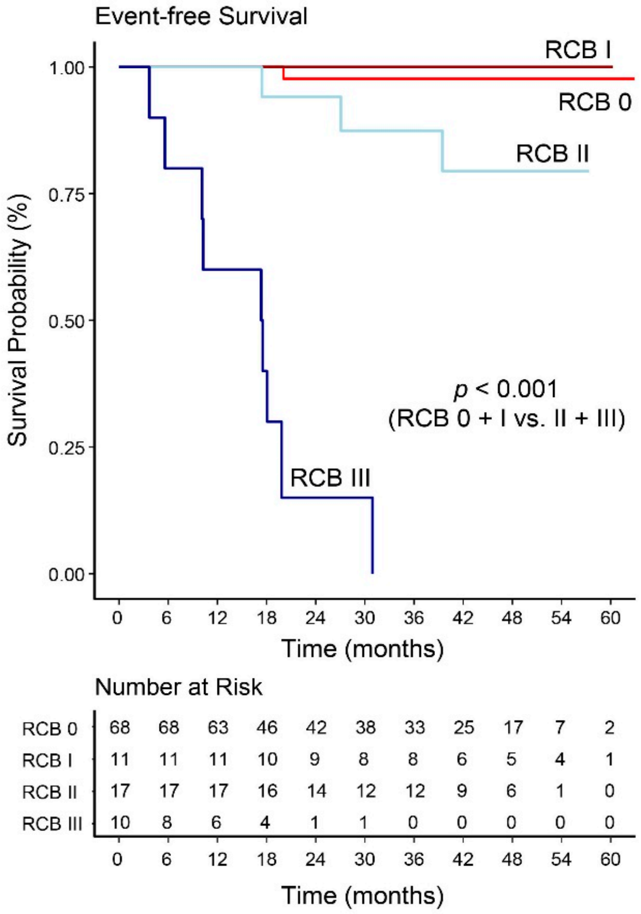

Supplementary Figure S2. Kaplan-Meier plots of event-free survival in high sTIL patients based on RCB groups.
sTIL, stromal tumor-infiltrating lymphocytes. RCB, residual cancer burden.
